# Zeolite-Based Poly(vinylidene fluoride) Ultrafiltration Membrane: Characterization and Molecular Weight Cut-Off Estimation with Support Vector Regression Modelling

**DOI:** 10.3390/membranes14040091

**Published:** 2024-04-16

**Authors:** Mieow Kee Chan, Syee Jia Tan, Andrew T. H. Yeow, Sok Choo Ng, Woei Jye Lau

**Affiliations:** 1 Centre for Water Research, Faculty of Engineering and the Built Environment, SEGi University, Petaling Jaya 47810, Malaysia; scm024228@segi4u.my; 2 Department of Chemical Engineering, Faculty of Engineering, Universiti Malaya, Kuala Lumpur 50603, Malaysia; andrew.yeow@um.edu.my; 3 Faculty of Arts and Science, International University of Malaya-Wales, Kuala Lumpur 50480, Malaysia; ashleyng@iumw.edu.my; 4 Advanced Membrane Technology Research Centre, Faculty of Chemical and Energy Engineering, Universiti Teknologi Malaysia, Skudai 81310, Malaysia; lwoeijye@utm.my

**Keywords:** dry–wet phase inversion, mechanical properties, poly(vinylidene fluoride), zeolite, support vector regression

## Abstract

Zeolite serves as a promising additive for enhancing the hydrophilicity of polymeric membranes, yet its utilization for bolstering the mechanical strength of the membrane remains limited. In this study, poly(vinylidene fluoride) (PVDF) membranes were modified by incorporating various concentrations of zeolite (0.5–2 wt%) to improve not only their mechanical properties, but also other features for water filtration. Membranes with and without zeolite incorporation were fabricated via a dry–wet phase inversion technique, followed by the application of a series of characterization techniques in order to study their morphological structure, mechanical strength, and hydrophilicity. The membrane filtration performance for each membrane was evaluated based on pure water flux and Bovine Serum Albumin (BSA) rejection. Field-Emission Scanning Electron Microscopy (FESEM) images revealed a dense, microvoid-free structure across all of the PVDF membranes, contributing to a high pristine PVDF membrane tensile strength of 14 MPa. The addition of 0.5 wt% zeolite significantly improved the tensile strength up to 19.4 MPa. Additionally, the incorporation of 1 wt% zeolite into PVDF membrane yielded improvements in membrane hydrophilicity (contact angle of 67.84°), pure water flux (63.49% increase), and high BSA rejection (95.76%) compared to pristine PVDF membranes. To further improve the characterization of the zeolite-modified PVDF membranes, the Support Vector Regression (SVR) model was adopted to estimate the molecular weight cut off (MWCO) of the membranes. A coefficient of determination (R^2^) value of 0.855 was obtained, suggesting that the SVR model predicted the MWCO accurately. The findings of this study showed that the utilization of zeolite is promising in enhancing both the mechanical properties and separation performance of PVDF membranes for application in ultrafiltration processes.

## 1. Introduction

Water scarcity is a pressing global issue due to the burgeoning of the human population, increases in industrialization, and extensive pollution from unintended discharges [1]. Hence, effective, efficient, and economically viable water reclamation and treatment technologies are of high significance to address this need. Polymer-based membrane separations have reached a notable level of maturity and practicality, owing to their exceptional capacity for removing pollutants efficiently and their straightforward setup and operational procedures [2]. Polyvinylidene fluoride (PVDF) is one of the most extensively used polymers in manufacturing microporous membranes due to its prominent properties, such as high mechanical strength, superior thermal and chemical resistance, and good membrane forming properties [3,4,5]. However, one of the most crucial problems in utilizing PVDF as a material for membrane fabrication is its hydrophobicity, which makes the membranes susceptible to surface fouling, thus reducing their separation efficiency and lifespan [6]. The inherent hydrophobicity of pristine PVDF membranes causes high adsorption rates of pollutants (such as BSA) onto their surface, forming an adsorption layer that leads to irreversible surface fouling [7]. Furthermore, PVDF membranes exhibit increased hydrophobicity after undergoing cleaning treatments such as sodium hypochlorite due to changes in the compaction of the membranes and surface fouling, as evidenced by an increase in the water contact angle of the membrane surface after cleaning treatments [8,9]. Hence, numerous improvements have been reported for the modification of PVDF membranes used in conventional pressure-driven separation processes to overcome their hydrophobicity, such as surface functionalization, surface coating, and blending of hydrophilic fillers [10,11,12,13].

PVDF membranes can be viably modified by blending hydrophilic porous materials such as zeolites and zeolite-based materials to form mixed matrix membranes (MMMs) [14,15]. Zeolites are crystalline aluminosilicate materials with microporous structures that exhibit exceptional molecular sieving properties. These attributes make them promising candidates for enhancing the performance of polymeric membranes through modification [16,17]. For example, it was found that the physical properties and separation performance of hollow fiber PVDF membranes fabricated through phase inversion were enhanced with the incorporation of silver-loaded sodium Y zeolites [18]. The zeolite-modified PVDF membranes exhibited a 141% improvement in pure water flux compared to pristine PVDF membranes and also showcased antibacterial capabilities. These enhancements were attributed to the microporous nature of zeolites and the incorporation of silver, respectively. In another study, Zhan et al. [19] modified polydimethylsiloxane (PDMS)/PVDF membranes using zeolite and found that at the composition of 30 wt% zeolite, the permeation flux of the membrane was significantly increased from 435.5 g/m^2^ h to 2993.8 g/m^2^ h, resulting in high separation factors for the pervaporation of ethanol/water mixture. Despite the appreciation in permeation and separation performances, one of the main drawbacks observed in such modification approaches is the relatively low mechanical integrity of zeolite-embedded membranes due to the conventional casting method employed, along with the formation of a thin skin layer and porous internal cross-section as the membrane structure [20]. This is supported by the works reported by Shi et al. [18] and Zhan et al. [19], in which the tensile strengths of the zeolite-incorporated PVDF membranes were within the ranges of 1.64–2.09 MPa and 1–2 MPa, respectively. These tensile strength values are relatively low.

Shi et al. [21] adopted a simple solvent evaporation technique for fabricating PVDF membranes on a nonwoven fabric support. This technique enabled the use of high zeolite loading (up to 90 wt%) to modify membranes and led to high ammonia treatment capacity (>850 L/m^2^), high turbidity, and high natural organic material rejection (~90%). Alternatively, a dry–wet phase inversion technique may be a viable method for fabricating MMMs that incorporate zeolite. The dry–wet phase inversion technique involves two stages of phase inversion: the initial solvent evaporation phase, followed by the exchange of solvent and non-solvent in the coagulation bath [22]. In the first stage of phase inversion (the dry phase), the casted film containing the solvent is left to partially evaporate, leading to the formation of a thin skin layer on the surface of the casted film. It is followed by the immersion of the casted film into a nonsolvent medium, causing the solvent–nonsolvent exchange to take place and develop polymeric films. In one study, this method yielded macrovoid-free membranes with a denser and thicker skin layer compared to conventional membranes made from wet phase inversion. In addition, the pore size in membranes can be controlled by manipulating the evaporation time and viscosity of the polymer solution [23,24].

In the characterization efforts of polymeric membranes, the molecular weight cut-off (MWCO) analysis is an important parameter in membrane classification, as it could represent the pore size of a membrane and its application. UF membrane has an MWCO close to 10 kDa, significantly larger compared to nanofiltration (NF) membranes, which exhibit MWCOs of between 200 and 2000 Da [25,26]. Traditionally, the MWCO of a membrane has been determined by exposing the membrane to various types of solutes with known solute sizes, such as polyethene glycol (PEG) and dextran individually. The concentration of the solute in the permeate was tested, and when 90% of the solute could be retained by the membrane, the MWCO of the membrane was identified. This method empirically determines the MWCO with a certain degree of accuracy; however, the experimental method is tedious, and it requires costly chemicals and reagents. An attempt has been made to simplify the MWCO experiment by using fluorescent silica nanoparticles as the feed [27]. Despite being claimed as a rapid quantitative measure for MWCO determination, this analysis method may be prohibitive due to the requirement of high-cost nanoparticles and an advanced nanoparticle tracking analyzer.

The use of machine learning (ML) in membrane modeling has gained significant research attention. The high predictive accuracy of ML enables it to be highly prospective in reducing the number of experiments that need to be conducted in membrane modeling. ML models and algorithms, particularly artificial neuron networks (ANNs), have garnered increasing levels of use in the modelling of membrane separation for wastewater treatment with high efficiency and reasonable accuracy [28]. This assists membrane researchers in estimating membrane performance with fewer experimental trials, enabling them to tailor membranes more accurately for specific applications [29].

In light of this, the objective of this study was to improve the hydrophilicity and tensile strength modified of PVDF membranes using conventional zeolites as the inorganic filler. The dry–wet phase inversion technique was used as a fabrication methodology to increase the tensile strength of the resultant membranes. The properties of the zeolite-modified PVDF membranes were then characterized in terms of morphological structure, mechanical strength, and hydrophilicity. Subsequently, water permeation tests were performed, and the solute rejection performances of the membranes were investigated. The functional groups present in the fabricated membranes were then determined through FTIR. To the best of our knowledge, no research has been performed on predicting the MWCO of a membrane using a machine learning approach. The Support Vector Regression (SVR) model was incorporated into this study to estimate the MWCO of a membrane. This model was chosen due to its effectiveness in addressing nonlinear problems and dealing with small sample sizes and high dimensions [28,29,30,31]. This work emphasized the fabrication of high-strength zeolite-incorporated PVDF membranes via use of the dry–wet phase inversion technique, and the use of an SVR model to estimate the molecular weight cutoff of membranes. The findings of this study may serve as a basis for membrane pore size prediction in future works.

## 2. Experimental Methods

### 2.1. Materials

PVDF pellets were purchased from Sanowart Group Co., Ltd. (Zibo City, China). PEG with a molecular weight (MW) of 20 kDa, and microporous zeolite 96096 of the 13X type (crystalline, <10 µm particle size), comprising silica (SiO_2_) and alumina (Al_2_O_3_) with metallic oxide, were purchased from Sigma Aldrich, Subang Jaya, Malaysia. N,N-dimethylformamide (DMF) was purchased from Fisher Scientific, Shah Alam, Malaysia. Egg albumin (EA) (MW = 44.3 kDa) was purchased from Sigma Aldrich, Malaysia, and bovine serum albumin (BSA) (MW = 66 kDa) and pepsin (MW = 35 kDa) were purchased from Acros Organics, Shah Alam, Malaysia.

### 2.2. Preparation of Polymer Solution

The polymer dope solution was prepared with an initial formulation of 16.5 wt% PVDF, 6 wt% PEG, and 77.5 wt% DMF. Various weight percentages of zeolite, ranging from 0.5% to 2%, were incorporated into the polymer solution composition as detailed in Table 1. The dope solution was prepared in a reaction flask by adding PVDF, PEG, and zeolite to DMF under constant vigorous stirring at 400 rpm for 4 h at 60 °C. Upon completion, the dope solution was left to sit at room temperature for 24 h to release air bubbles.

### 2.3. Membrane Fabrication by Dry–Wet Phase Inversion Method

The membrane was fabricated by casting the dope solution at a predetermined thickness of 200 µm using an automated casting machine [32]. The membrane was developed using a dry–wet phase inversion method, where the casted solution was firstly placed into the oven to be dried for 9 min at 60 °C, followed by immersion into a coagulation bath (RO water) for phase exchange to occur. The casted membrane was then collected and subjected to thorough RO water rinsing to remove any residual solvent. Finally, the membrane was kept in a container filled with RO water.

### 2.4. Membrane Characterisation Methods

#### 2.4.1. Fourier Transform Infrared (FTIR) Spectroscopy

The functional groups of the PVDF membranes were observed through attenuated total reflectance-Fourier transform infrared spectroscopy (ATR-FTIR) performed on a Perkin Elmer Spectrum 100 FTIR spectrometer (Perkin-Elmer Inc., Norwalk, CT, USA). The FTIR spectra of the PVDF membranes were obtained over the wavenumber range of 4000–600 cm^−1^, averaging four repeated scans for each measurement. The total area of the FTIR spectra peaks were determined and calculated using the accompanying Perkin Elmer Spectrum software (Version 6.3.5).

#### 2.4.2. Field Emission Scanning Electron Microscopy (FESEM) Imaging

The morphology and structure of the PVDF membranes with and without modification were examined using FESEM imaging (Zeiss Supra 40, Jena, Germany). The cross-sectional area of the membranes was obtained by freeze-fracturing the samples using liquid nitrogen. Subsequently, cross-sectional areas of the membranes were coated with a thin, conductive gold layer and placed on an imaging stub lined with carbon tape. The FESEM images were captured at magnifications of 1000× and 3000× with varying accelerating voltages (10–15 kV) to ensure image clarity. Additionally, the membrane thickness was measured and reported through FESEM imaging.

#### 2.4.3. Membrane Contact Angle Analysis

The surface contact angle of the PVDF membranes was measured using the Attension Theta Lite Optical Tensiometer (Biolin Scientific, Stockholm, Sweden) with the accompanying OneAttension software (Version 1.7). Ten measurements were taken at random, non-repeated spots of the same membrane sample in order to yield the mean value and standard deviation.

#### 2.4.4. Pure Water Flux (PWF) Analysis

The pure water flux (PWF) of the zeolite-modified PVDF membranes was determined using a 50 mL dead-end filtration cell unit (Amicon Stirred UF Cell, Millipore Corporation, Temecula, CA, USA) with an effective membrane area of 1.5904 × 10^−3^ m^2^. This analysis was conducted by applying nitrogen gas across the membrane at 2.5 bar, and the permeate volume was collected and recorded over a duration of 30 min. Equation (1) was employed to calculate the PWF (*J*, L/m^2^·h·bar) of the membrane:

(1) $$\;J=\frac{Q}{A\;\times △P}$$

where *Q* is the flow water of the permeate (L/h), *A* is the effective area of the membrane (m^2^), and △*P* is the operating pressure (bar).

#### 2.4.5. Solute Rejection and Molecular Weight Cut-Off (MWCO) Analysis

The solute rejection was characterized using 1 g/L protein solution (Pepsin, EA and BSA) after the PWF tests. The experiment was carried out in ascending order of solute MW to minimize pore blockage during filtration using the same circular piece of membrane. The protein solutions were prepared via dissolving appropriate amounts of protein in RO water, which has a recorded pH between 6.0 and 6.5. The membranes were backwashed with RO water before proceeding to the next solute rejection test in order to minimize the fouling effect on the membrane separation. The volume of permeate was recorded after a filtration duration of 30 min, and the permeate was sampled into a quartz cuvette to measure the solute concentration using a UV-visible spectrophotometer (Shimadzu UV-1900i, Shimadzu Corporation, Kyoto, Japan) at an absorbance wavelength of 280 nm. The solute concentration was determined based on a calibration graph produced from known protein solution concentrations, and the solute rejection (*R*, %) was calculated using Equation (2):

(2) $$R\;\left(\%\right)=\frac{C_{f}-C_{p}}{C_{p}}\times 100\%$$

where *C_f_* is the solute concentration of the feed solution, and *C_p_* is the solute concentration of the permeate solution. The MWCO analysis was conducted by plotting solute rejection against the ascending sequence of the solute’s molecular weight, and the MWCO of the membranes was determined graphically at 90% solute rejection.

#### 2.4.6. Machine Learning Model for MWCO Prediction

Approximately 150 datapoints were extracted from the MWCO graph, and the ranges of the data are tabulated in Table 2. The dataset was split into the training set and the testing set at a ratio of 60:40. In other words, the 150 datapoints (inclusive of 60% of PVDF-1) extracted from the MWCO graph were used to train the model, while 40% of the PVDF-1 data (testing set) were used to evaluate the performance of the model. This 40% of datapoints obtained from the laboratory experiment were to be compared to the predicted MWCO values generated by the machine learning model. To observe better performance of the model, additional train–test sets with respective ratios of 70:30 and 80:20 were prepared for the development of the SVR model. The training set was used to train the model, while the testing set was used to evaluate the performance of the model. Zeolite concentration and MWCO served as the inputs for the SVR model, while the rejection rate was the output. The predicted value of the model was then validated using 42 sets of data. The predictive performance of the model was measured in terms of *R*^2^ and mean square error (*MSE*), as shown in Equations (3) and (4), respectively.

(3) $$R^{2}=1-\frac{{\sum_{i}\left(y_{i}-{\hat{y}}_{i}\right)}^{2}}{\sum_{i}{\left(y_{i}-\bar{y}\right)}^{2}}$$

(4) $$MSE=\frac{\sum_{i=1}^{N}(y_{i}-\overset{\wedge }{y_{i}}{)}^{2}}{N}$$

where $y_{i}$ and ${\hat{y}}_{i}$ are the actual and predicted output values, respectively, and N is the number of data sets.

#### 2.4.7. Mechanical Properties

The tensile strength and elongation tests for the PVDF membranes were performed using a Shimadzu AG-X Universal Tensile Machine (Shimadzu Corporation, Kyoto, Japan) according to the ASTM D882-12 standard [33]. Ten dry samples of each membrane formulation were cut vertically at a measurement of 1.35 cm by 13.5 cm (1:10 ratio). The membrane samples were secured at both ends, ensuring a gauge length of 10 cm, and then subjected to a pulling rate of 0.5 mm/min with a 5 kN load. Ten readings were measured, and the average value was reported. The tensile strength (MPa) and elongation percentage (ϵ, %) at fracture of the membranes were calculated using Equations (5) and (6), respectively, according to the ASTM D882-12 standard.

(5) $$Tensile\;Strength=\frac{Load\;at\;break\;}{\left(Original\;width\right)\times (Original\;thickness)}$$

(6) $$\;\;\epsilon =\frac{Elongation\;at\;fracture\;}{Initial\;gauge\;length}\times 100$$

## 3. Results and Discussion

### 3.1. FTIR Spectra of Zeolite-Modified PVDF Membranes

**Figure 1:**
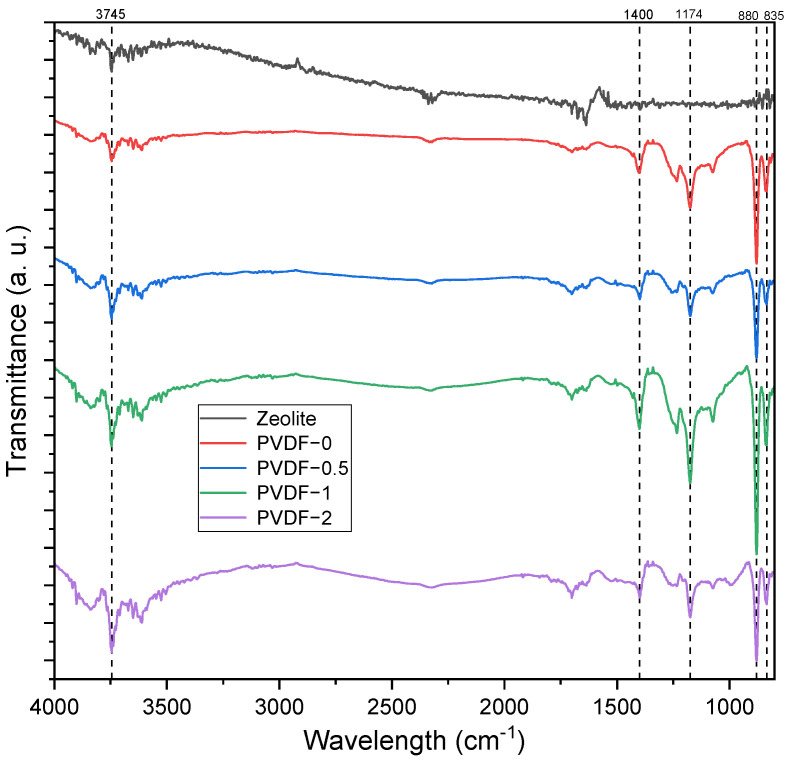
FTIR spectra of the zeolite, pristine PVDF membrane, and zeolite-modified PVDF membranes.

The FTIR spectra of zeolite, pristine PVDF membrane (PVDF-0), and zeolite-modified PVDF membranes are presented in Figure 1, whereas the band assignment and calculated peak area data of the respective samples are presented in Table 3. In general, the membrane samples exhibited similar peaks at wavelengths of 1174 cm^−1^ and 879 cm^−1^, both of which are ascribed to the organic structure of PVDF. The band of 1174 cm^−1^ was ascribed to the C–C group, while the 879 cm^−1^ band was ascribed to the C–C–C asymmetrical stretching vibration. The peak at 835 cm^−1^ was attributed to the C–F stretching vibration in the PVDF polymer. The characteristic absorption peak of zeolite at 1636.63 cm^−1^, associated with the bonding of hydrogen to the lattice oxygen, was absent in the resulting membranes. The total area of the peak within the range of 1584.45–1789.55 cm^−1^ was further analyzed, and it was revealed that the total area increased as the concentration of zeolite increased up to 1 wt%. The observation of this peak revealed the highest total area at 40,875.65% T·cm^−1^ and supported the results of the contact angle measurements of the PVDF-1 membranes, based on the theory of increase in free vibrating water molecules. Additionally, an increase in band intensity was observed in the modified PVDF membrane at 3745 cm^−1^, which was ascribed to the presence of O–H group interactions. This shows that the incorporation of zeolite into the PVDF matrix may enhance the hydrophilicity of the membrane surface. Due to the lower significant differences of the O–H band (as seen in Figure 1), the total area of the peak within the wavelengths of 3500–3903.5 cm^−1^ was determined and is presented in Table 3.

### 3.2. Membrane Morphology

**Figure 2:**
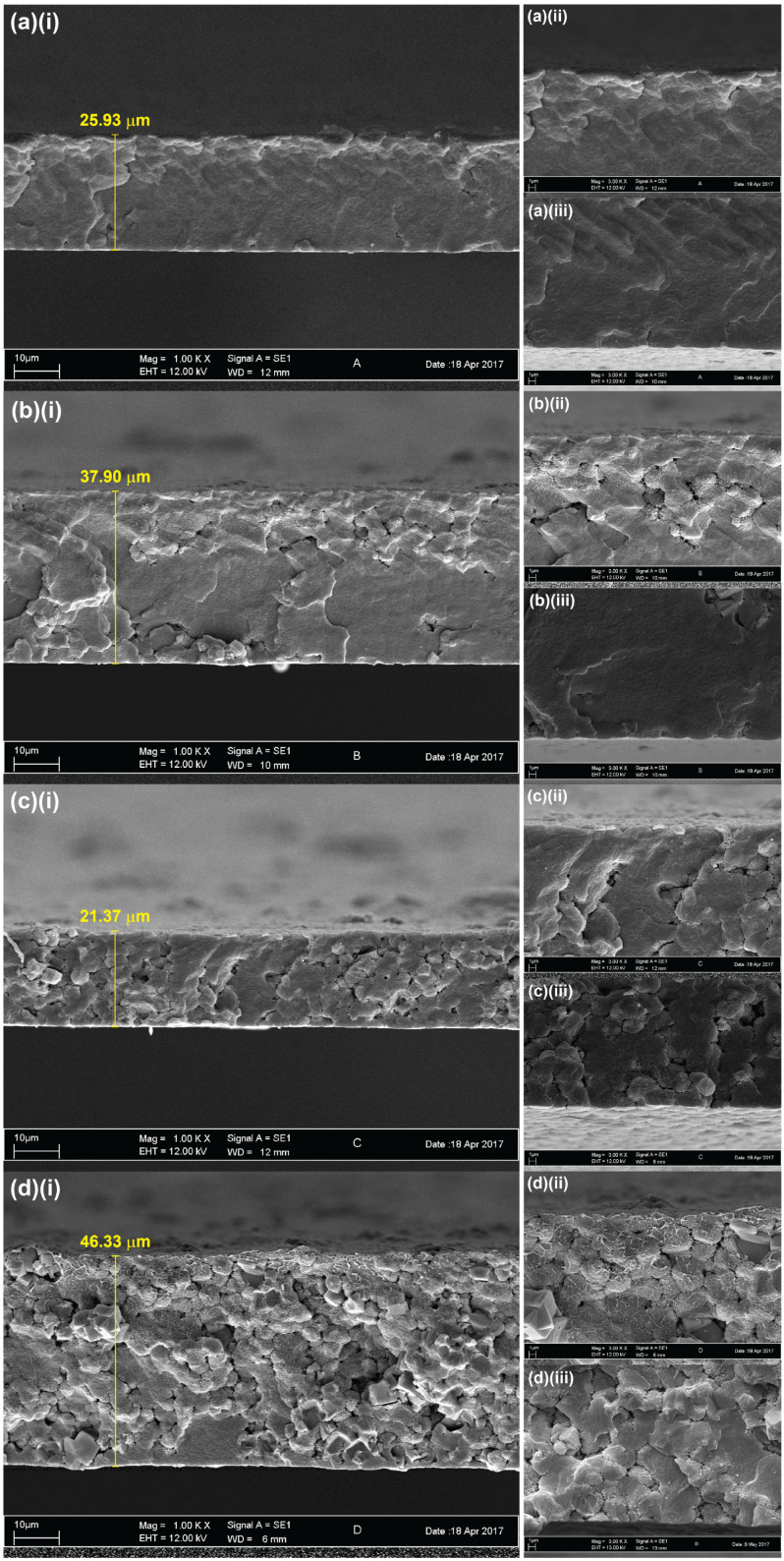
FESEM micrographs of (**a**) PVDF-0, (**b**) PVDF-0.5, (**c**) PVDF-1, and (**d**) PVDF-2 membranes at magnifications of (**i**) 1000×, (**ii**) 3000× near the surface of the membrane, and (**iii**) 3000× cross-section.

The FESEM micrographs revealing the cross-section of the pristine and zeolite-modified PVDF membranes are presented in Figure 2, along with the measured thickness values. The membrane morphologies across all samples exhibited uniformly dense structures without any noticeable microvoids. Additionally, the membranes appeared to become increasingly porous with the increased loading of zeolite, which may be attributed to the intrinsic porous characteristics of zeolite. A previous study has reported that an increased zeolite concentration increases pore sizes in polyethersulfone-based membranes [34]. The membrane structure observed in the PVDF-2 membrane appeared to be more disordered than the membranes containing lower zeolite loadings (PVDF-0.5 and PVDF-1), and the cubic structures of the embedded zeolites in the PVDF-2 membranes were clearly visible. This disparity may be attributed to the pronounced aggregation of zeolite particles at higher concentrations. Previous studies have shown that the aggregation caused by excessive use of inorganic additives could lead to pore blockages, which reduce membrane permeation [35,36]. Hence, the optimal concentration of additives should be considered in the production of high performance membranes.

### 3.3. Contact Angle and Pure Water Flux Analysis

**Figure 3:**
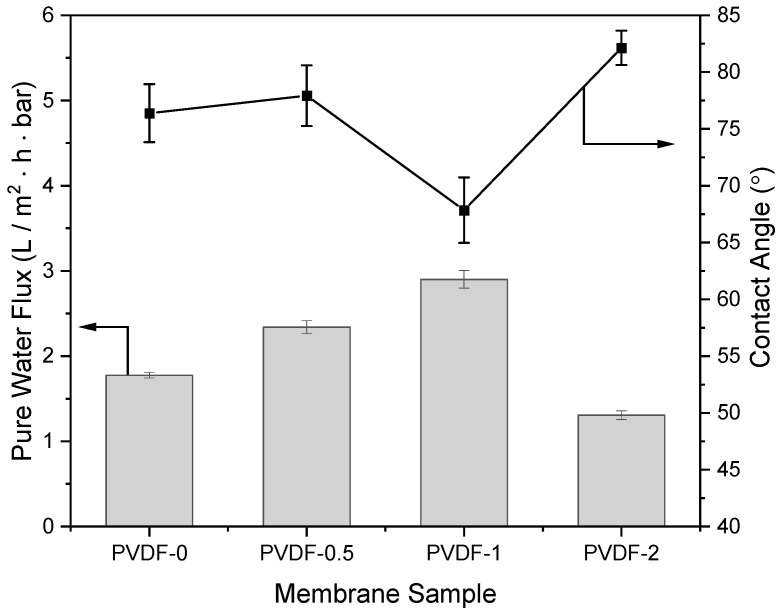
Pure water flux (PWF) and water contact angle of zeolite-modified PVDF membranes.

The contact angle of zeolite-modified PVDF membranes is presented in Figure 3, along with the PWF analyses. The addition of 1 wt% zeolite to the PVDF membrane (PVDF-1) resulted in a decrease in contact angle from 76.38°, as observed in the pristine PVDF membrane (PVDF-0), to 67.84°. This indicates the enhancement of surface wettability and hydrophilicity in modified PVDF membranes, which is proven by the FTIR spectra in Figure 1, where the presence of an O–H group was observed in both PVDF-0.5 and PVDF-1, in comparison to PVDF-0. The total areas of the O-H groups in the range of 3500 to 3903.5 cm^−1^ were 40,742.91% T·cm^−1^ and 40,875.65% T·cm^−1^ for PVDF-0.5 and PVDF-1, respectively, as tabulated in Table 3. Furthermore, the PWF of the pristine PVDF membrane was enhanced in PVDF-1, exhibiting a notable increase of 63.49%, rising from 1.77 to 2.90 L/m^2^·bar·h. The PVDF-0.5 membrane also exhibited a 31.85% increase in PWF, suggesting the synergistic effect of zeolite as a filler in the membrane matrix. However, it was observed that when 2 wt% zeolite was added to the membrane (PVDF-2), it caused an adverse effect on the membrane characteristics by increasing its contact angle, resulting in the hydrophilicity of the membrane being reduced, and thus reducing its PWF. Supported by findings from FESEM imaging, the detrimental effects observed in PVDF-2 could be attributed to the membrane’s thickness and filler overloading and agglomeration, leading to pore blockages and increased surface resistances. Similar findings were reported by Anis et al. [37], where varied wt% of nano zeolite-Y was incorporated into poly(vinyl) alcohol-networked cellulose membranes and applied for reverse osmosis. At 0.5 wt% nano zeolite-Y loading, the highest flux of 5.1 L/m^2^·h was reported at 25 bar pressure, yielding a high salt rejection percentage of 99.52%. The addition of nano zeolite-Y to 1.0 wt% increased its contact angle and reduced the flux to 2.7 L/m^2^. The overloading of nanoparticles, such as nano-iron oxides, in PVDF membranes was similarly shown to reduce the membrane contact angle and PWF [38].

### 3.4. Solute Rejection Performance

**Figure 4:**
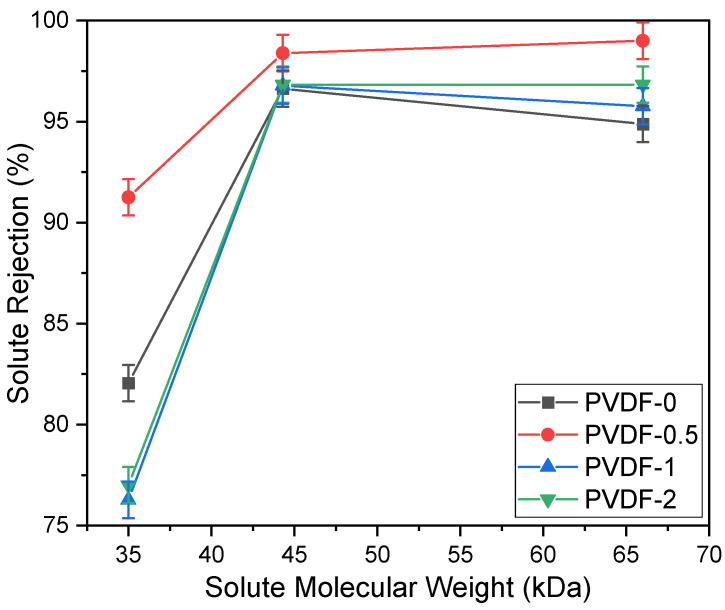
Solute rejection of PVDF membranes incorporated with different concentrations of zeolite, plotted against solute molecular weight (pepsin, EA, and BSA).

The solute rejection performance of PVDF membranes incorporated with different concentrations of zeolite are presented in Figure 4. In terms of solute removal rate, the PVDF-0.5 membrane exhibited the highest solute rejection percentage compared to the other membranes, recording 91.25%, 98.39%, and 99% rejection for pepsin, EA, and BSA, respectively. As membrane pore sizes are directly proportional to the MWCO, it was determined in this study that the MWCO values of the PVDF-1 and PVDF-2 membranes were the highest at 40 kDa, while the PVDF-0.5 membrane exhibited a MWCO of 35 kDa. Based on the solute rejection performance, it can be inferred that the surface charge of the PVDF membranes are altered to become slightly negative when embedded with negative zeta potential additives such as zeolite, leading to eventual repulsion of negatively charged solutes (pepsin, EA, and BSA) and resulting in high solute rejection [18]. Additionally, the dense structure of the PVDF membranes lead to significant resistance in solute permeation, despite achieving a high rejection rate, as a result of the fabrication method utilized in this study.

### 3.5. Prediction of MWCO of Zeolite-Modified Membrane Using SVR Model

**Figure 5:**
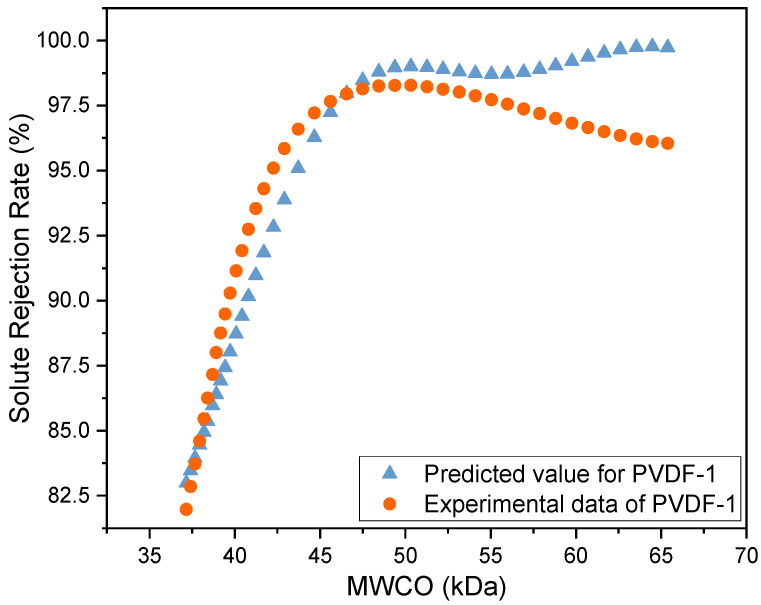
Experimental and predicted (SVR model) MWCO for the zeolite-modified PVDF-1 membrane.

Figure 5 shows the predicted MWCO of the PVDF-1 membrane, determined using the SVR model, and compares it with the MWCO values of the PVDF membranes as obtained from the filtration experiment. The predicted solute rejection of the PVDF-1 membrane increased as the MWCO of the solutes increased. Minimum changes were observed in the rejection rate when the solute size was increased beyond 50 kDa. This trend is similar to that observed in the experimental data for the other zeolite-modified PVDF membranes. The mean square error of the model was 3.59, and its R^2^ value was 0.855. Table 4 shows the comparison of the SVR model with different train–test sets. From observation, the range of R^2^ values was between 0.8558 and 0.9139. In controlled environments, the variance across predictive measurements is likely to be low, and therefore R^2^ values can be expected to lie in the 0.8 range [39]. The R^2^ value achieved in this study suggests that the model provides a good fit to the data, with a larger proportion of the variance in the rejection rate being associated with the MWCO. Similarly, P Fernandes et al. [40] and Islam Khan et al. [41] reported similar ranges in R^2^ value for some of their regression models. This can be attributed to the ability of the SVR model to manage non-linear data, as it operates by first mapping the original feature space into a higher-dimensional space using a kernel function [42,43]. It then identifies the hyperplane in this transformed space that best fits the data while maximizing the margin between the hyperplane and the closest data points to minimize training data error. This method is particularly beneficial for dealing with small sample sizes and high dimensions [31]. The MWCO of the PVDF-1 membrane was 39.73 kDa when it was estimated from the predicted curve, as shown in Figure 5. This is very close to the MWCO that was obtained from the experimental data, which was 40 kDa, indicating good predictive performance of the SVR model and initial demonstrability of application in the prediction of MWCO for polymeric membranes.

### 3.6. Mechanical Properties of Zeolite-Modified Membranes

**Figure 6:**
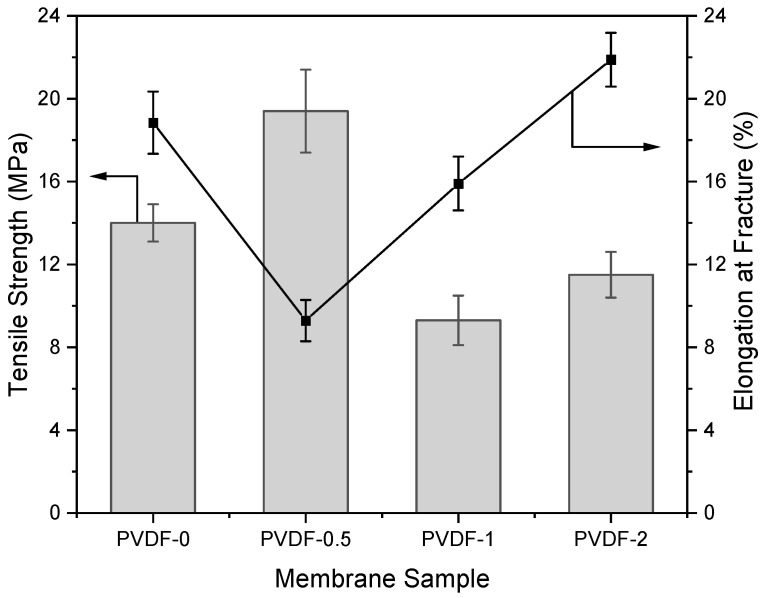
Tensile strength and elongation percentage at fracture of zeolite-modified PVDF membranes.

The mechanical properties of the zeolite-modified PVDF membranes were inferred based on their tensile strength and elongation percentages at fracture, as shown in Figure 6. The findings show that the tensile strength of the PVDF membrane reached its peak when incorporating a composition of 0.5 wt% zeolite, resulting in a 38.57% improvement in tensile strength from 14 MPa to 19.4 MPa. This enhancement could be attributed to increased interfacial interactions between the polymer chain and the additives, facilitated by the addition of zeolite as the inorganic filler [44,45]. On top of the increase in tensile strength, the elongation at fracture was reduced from 18.84% to 9.29%, indicating an increase in the membrane rigidity. However, a decrease in PVDF membrane tensile strength was observed at higher zeolite loading of 1–2 wt%; this could be explained by filler overloading, reducing the intermolecular interactions among the polymer chains. The membranes fabricated in this study have reportedly higher overall tensile strength than prior zeolite-incorporated PVDF membranes. For instance, in one study, nanocomposite PVDF membranes incorporated with zeolites (ZSM-5 particles) were fabricated through electrospinning and copolymer crosslinking for battery separator applications, exhibiting tensile strengths of up to 3.2 MPa [46]. In another study, PVDF membranes were loaded with 5–25 wt% of zeolitic imidazolate framework (ZIF-8) nanoparticles and fabricated using a non-solvent induced phase inversion technique, in which 10 wt% of nanoparticle loading yielded PVDF membranes with the highest strength of 3.25 MPa [47]. Therefore, this observation suggests the significance of trade-off between membrane permeability and tensile strength [48,49]. Based on the experimental results, the PVDF-1 membrane exhibited substantial enhancements in membrane hydrophilicity, PWF, and BSA rejection. However, it also exhibited lower tensile strength compared to the pristine PVDF membrane.

## 4. Conclusions

High tensile strength PVDF membranes were fabricated via the dry–wet phase inversion method and modified by incorporating compositions of zeolite from 0.5–2 wt%. From this study, the incorporation of 1 wt% zeolite into PVDF membrane yielded significant improvements in membrane hydrophilicity (contact angle reduction from 76.38° to 67.84°), PWF (63.49% increase), and BSA rejection (95.76%) compared to pristine PVDF membrane. The dry–wet phase inversion technique yielded PVDF membranes with dense, microvoid-free structures across all samples, resulting in membranes with high tensile strength (14 MPa). Furthermore, zeolite-modified PVDF membranes exhibited denser and thicker skin layers, and the incorporation of 0.5 wt% zeolite yielded PVDF membranes with the highest recorded tensile strength of 19.4 MPa. However, the addition of 1 wt% zeolite reduced the tensile strength to 9.3 MPa, which may be attributed to a trade-off between permeability and strength as a result of the alterations in the membrane’s morphology. The MWCO of PVDF-1 predicted from the SVR modelling was only 0.07% smaller than the MWCO determined from experimental data. This indicated the accuracy of the developed model in MWCO prediction. The fabrication and modification of PVDF membranes using the dry–wet phase inversion method and zeolite incorporation, respectively, presents a viable method to enhance the mechanical properties and separation performances of PVDF membranes.

## Figures and Tables

**Table 1:** Composition of polymer solution.

Membrane Designation	PVDF (wt%)	PEG (wt%)	DMF (wt%)	Zeolite (wt%)
PVDF-0	16.5	6	77.5	0
PVDF-0.5	16.5	6	77	0.5
PVDF-1	16.5	6	76.5	1
PVDF-2	16.5	6	75.5	2

**Table 2:** Extracted data for SVR modelling.

Zeolite Concentration (wt%)	MWCO (X)	Rejection Rate, % (Y)	Number of Data
0.0	35,108–65,634	82–97	42
0.5	35,541–66,794	91–100	34
1.0	35,142–46,552	76–98	29
2.0	35,224–64,517	78–98	47

**Table 3:** FTIR analysis of PVDF membranes fabricated at zeolite concentrations of 0.5, 1, and 2 wt%.

Compound	Characteristic Peak (cm^−1^)	Band Assignment	Total Area within the Range of 1584.45 cm^−1^ to 1789.55 cm^−1^ (O-H) Lattice Water (%T·cm^−1^)	Total Area within the Range of 3500 cm^−1^ to 3903.5 cm^−1^ (O-H Group) (%T·cm^−1^)
Zeolite	1636.63	(O–H) lattice water	20,528.68	-
PVDF-0	1174.52	C–C group		
	879.71	C–C–C asymmetrical stretching vibration	20,538.65	-
PVDF-0.5	3745.36	O–H group		
	1174.30	C–C group	20,714.14	40,742.91
	879.62	C–C–C asymmetrical stretching vibration		
PVDF-1	3745.47	O–H group		
	1399.86	CH_2_ wagging vibration		
	1174.72	C–C group		
	879.52	C–C–C asymmetrical stretching vibration	20,827.06	40,875.65
	835.95	C–F stretching vibration		
PVDF-2	3745.01	O–H group		
	1174.43	C–C group		
	879.59	C–C–C asymmetrical stretching vibration	20,528.68	40,441.58
	835.74	C–F stretching vibration		

**Table 4:** Comparison of SVR model with different train–test sets.

Training-Testing Dataset Ratio	R^2^	MSE
60:40	0.8548	3.5885
70:30	0.8796	2.9759
80:20	0.9139	2.1277

## Data Availability

The original contributions presented in the study are included in the article, further inquiries can be directed to the corresponding author.
